# Stepwise Provisional *versus* Planned Double Stenting Strategies in Treating Unprotected Left Main Distal Bifurcation Lesions: A Systematic Review and Meta-Analysis Comprising 11,672 Patients

**DOI:** 10.31083/j.rcm2408216

**Published:** 2023-07-31

**Authors:** Dongdong Li, Hao Liu, Chuncheng Gao, Jing Liu, Pengyun Liu, Miaomiao Cheng, Qiangsun Zheng, Jie Deng, Mingming Zhang, Zhonghua Luo, Wangang Guo

**Affiliations:** ^1^Department of Cardiology, Tangdu Hospital, Air Force Medical University, 710038 Xi'an, Shaanxi, China; ^2^Department of Cardiology, The Second Affiliated Hospital of Xi'an Jiaotong University, 710004 Xi'an, Shaanxi, China; ^3^Department of Intervention Radiology, Tangdu Hospital, Air Force Medical University, 710038 Xi'an, Shaanxi, China

**Keywords:** bifurcation, double stenting, left main, MACE, provisional stenting

## Abstract

**Background:**

Provisional stenting is the preferred strategy for non-left main bifurcation lesions. However, its superiority over planned double stenting for unprotected left main distal bifurcation (UPLMB) lesions remains unclear. Previous studies have reported conflicting results.

**Methods:**

Randomised controlled trials (RCTs) and observational studies comparing the outcomes of provisional stenting to planned double stenting for UPLMB lesions were identified. The primary endpoint was major adverse cardiac events (MACE). The secondary endpoints were myocardial infarction (MI), target vessel revascularisation (TVR), target lesion revascularisation (TLR), all-cause death, cardiac death and stent thrombosis (ST). Aggregated odds ratios (OR) and 95% confidence intervals were calculated. A sensitivity analysis was conducted if *$I^{2}$* was >50% or *p* < 0.01. Publication bias analysis was considered if more than 10 studies were enrolled.

**Results:**

Two RCTs and 19 observational studies comprising 11,672 patients were enrolled. Provisional stenting had a significantly lower incidence of MACE, mainly driven by TLR and TVR. Double stenting had a significantly lower incidence of cardiac death. In addition, patients undergoing provisional stenting had a lower tendency towards the occurrence of MI, while patients undergoing double stenting had a lower tendency towards all-cause death and ST.

**Conclusions:**

A provisional stenting strategy was associated with lower MACE, TVR and TLR but higher cardiac death. Further investigation is needed through RCTs to assess which strategy performs better.

## 1. Introduction

An unprotected left main distal bifurcation (UPLMB) lesion is a lesion that involves the distal bifurcation of the left main (LM) coronary artery [1, 2]. It remains one of the most challenging lesions in the field of cardiac interventional therapy because of its unique anatomical location and geometry [3]. LM lesions include protected and unprotected lesions based on the presence of blood supply from the vascular bridge or good collateral circulation from the right coronary artery. Among all types of coronary artery lesions, UPLMB has the worst prognosis. Currently, there are two percutaneous coronary intervention (PCI) strategies for UPLMB lesions: stepwise provisional stenting and planned double stenting. The stepwise provisional stenting strategy involves placing stents in the main vessel crossing over the side branch and another stent, if necessary, in the branch vessel. The planned double stenting strategy involves placing stents both in the main vessel and the branch vessels. The former has been proven to be the preferred strategy for non-LM bifurcation lesions [4]. However, controversy still remains regarding which strategy is superior for UPLMB lesions. There have only been two multicentre randomised controlled trials (RCTs) addressing this issue, and they drew conflicting conclusions. In the DKCRUSH-V Registry, Chen *et al*. [5] concluded that provisional stenting increased the rate of target lesion revascularisation failure (TLF) and stent thrombosis (ST) over three years of follow-up. In contrast, the European Bifurcation Club Left Main (EBCLM) trial proved that provisional stenting had a lower rate of major adverse cardiac events (MACE) [6]. Other observational cohort studies have also not come to consistent conclusions. Therefore, we performed this systematic review and meta-analysis to clarify which of the two interventional strategies was superior. We also compared the long-term outcomes in the drug-eluting stent (DES) era with the goal to provide convincing data-based medical evidence for selecting the best PCI plan for UPLMB patients.

## 2. Methods

### 2.1 Literature Searching

A comprehensive search was conducted using PubMed, Embase, Ovid Medline, Cochrane Database, Web of science, CNKI and ClinicalTrails.gov. RCTs and observational studies comparing provisional and planned double stenting for distal UPLMB disease published from library or database construction to 1 Jan. 2023, were searched. The key search terms included “left main”, “provisional”, “double”, “one”, “two”, “simple” and “complex”. The search terms were retrieved using a free combination method, and all relevant references were evaluated for additional studies that were not identified from the initial database searches. The search strategy is presented in **Supplementary Table 1**. This study was conducted in accordance with the Preferred Reporting Items for Systematic Reviews and Meta-Analyses statement (**Supplementary Table 2**).

### 2.2 Literature Inclusion and Exclusion Criteria

Inclusion criteria were: (1) RCTs and observational studies comparing provisional stenting and planned double stenting strategies for distal UPLMB disease; (2) comparable general information between the two strategies; (3) DES stents used in both strategies; and (4) outcome indicators including at least one of MACE, all-cause death, cardiac death, myocardial infarction (MI), target vessel revascularisation (TVR), target lesion revascularisation (TLR), or ST. Exclusion criteria were: (1) incomplete or ambiguous data; (2) follow-up period of less than 6 months; and (3) studies that shared the same participants.

### 2.3 Data Extraction

Two reviewers in the research group (DL and HL) independently screened the retrieved literature and extracted information. In case of disagreement of the status of the study, it was resolved through discussion with a third reviewer (CG). The extracted data included: (1) basic information of the enrolled studies, including first author, publication year, follow-up period, and study type; (2) general data of participants, including sample size, mean age, gender ratio, ethnicity, clinical diagnosis, medication, and lesion characteristics; (3) PCI strategy, including provisional, T, V, Y, Crush, double kissing technique (DK)-Crush, culotte, etc*.*; (4) outcome indicators, including all-cause death, cardiac death, MI, TLR, TVR, ST, and MACE; and (5) other information such as stent type and number, intravascular ultrasound (IVUS), and proximal optimal technique (POT).

### 2.4 Outcomes and Definitions

The primary endpoint of this meta-analysis was MACE, defined as a composite of death, MI and TLR/TVR. The composition varied among the enrolled studies, and this review adopted the initial definition of the studies. In some articles, MACE is defined as TLF. The secondary endpoints were ST and the individual components of the primary endpoint, including all-cause death, cardiac death, MI, TLR, TVR, and ST. The definitions of every endpoint in each study are summarized in **Supplementary Table 3**.

### 2.5 Risk Assessment of Bias

DL and HL conducted bias risk assessment. CG resolved any disparity by arbitration. RCTs were assessed by the Cochrane Collaboration tool 5.3 (the Cochrane Collaboration, Copenhagen, Denmark) [7], while observational studies were assessed by the Newcastle-Ottawa Quality Assessment Scale (NOS) [8].

### 2.6 Statistical Analysis

STATA/MP 17.0 (Stata Corporation, College Station, TX, USA) was used to calculate aggregated odds ratios (OR) at 95% confidence intervals. Heterogeneity between the studies was explored using the $I^{2}$ test and the fixed-effects model was used when *p* > 0.01 and $I^{2}$ < 50%, while the random-effects model was used if not. A heterogeneity test and sensitivity analysis were used to select the origin of heterogeneity. Contour-enhanced funnel plots, a regression-based Egger test, and non-parametric trim-and-fill analysis were used to assess publication bias if the number of studies was more than 10. *p*-value < 5% was considered the difference was significant.

## 3. Results

### 3.1 Searching Results and Baseline Information 

**Fig. 1.**
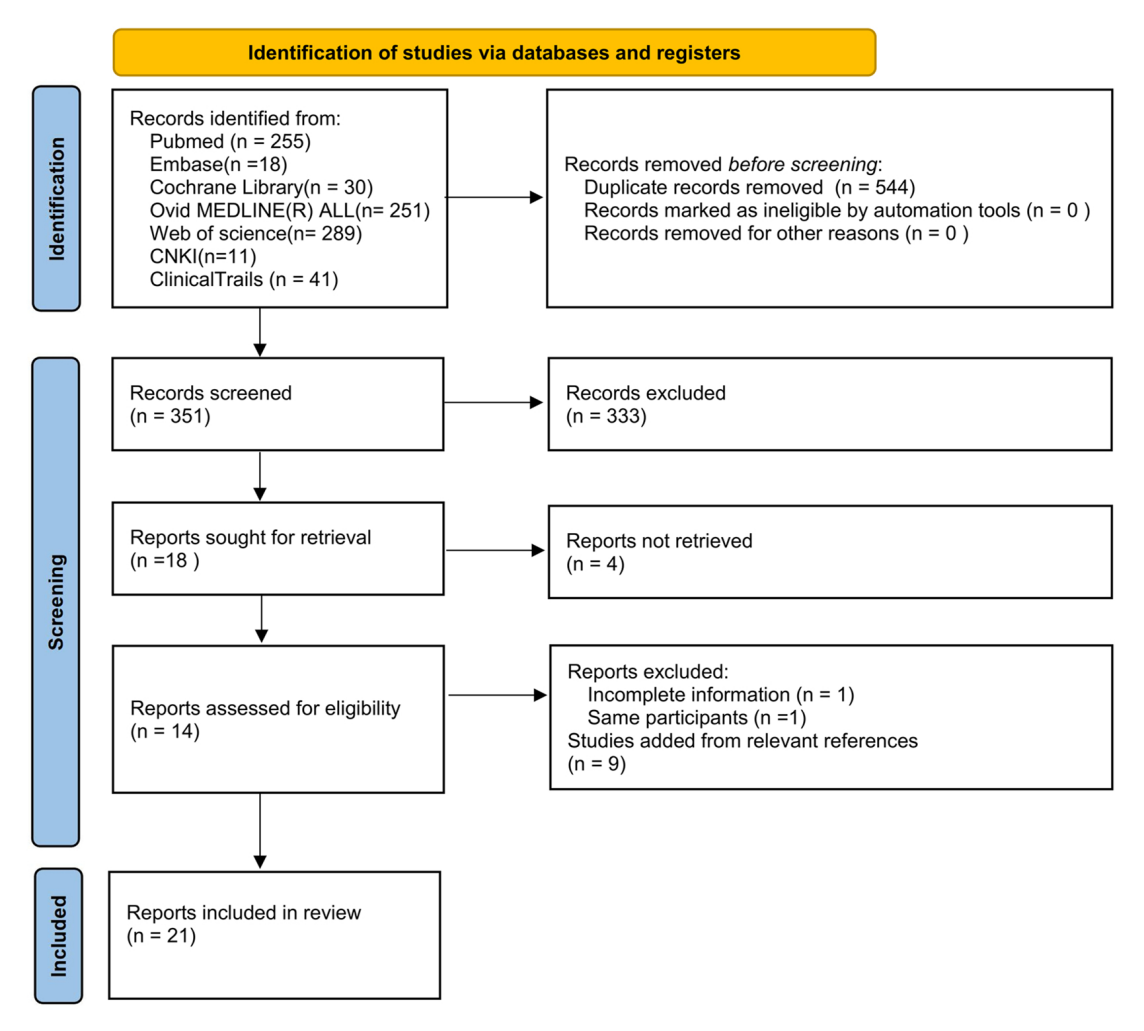
**Literature retrieval process**.

Fig. 1 describes the flowchart that was employed to identify qualifying studies for this meta-analysis. Six databases and ClinicalTrails.gov were searched. From 921 identified studies, 570 were excluded for being duplicates, 333 for not meeting the inclusion criteria, four for not being retrievable, and two for meeting the exclusion criteria. Nine were added through reviewing the relevant references. Finally, 21 studies were enrolled [5, 6, 9, 10, 11, 12, 13, 14, 15, 16, 17, 18, 19, 20, 21, 22, 23, 24, 25, 26, 27]. Nineteen studies had data on MACE [5, 6, 9, 10, 11, 13, 14, 15, 16, 17, 18, 19, 20, 21, 23, 24, 25, 26, 27], 18 had data on MI [5, 6, 10, 11, 12, 13, 14, 15, 16, 17, 20, 21, 22, 23, 24, 25, 26, 27], 17 had data on TLR [5, 6, 9, 11, 12, 13, 14, 15, 16, 17, 18, 19, 20, 23, 24, 25, 26], six had data on TVR [10, 11, 13, 15, 21, 27], 16 had data on ST [5, 6, 10, 11, 13, 15, 16, 18, 19, 20, 21, 22, 23, 25, 26, 27], 11 had data on cardiac death [5, 13, 15, 16, 17, 19, 20, 21, 22, 24, 25], and 14 had data on all-cause death [6, 9, 10, 11, 12, 13, 14, 19, 20, 22, 23, 24, 25, 27]. The studies were performed from 2002 to 2019, and the publication years ranged from 2006 to 2022. A total of 11,672 patients were enrolled in the study. The general characteristics of the studies are listed in Table 1 (Ref. [5, 6, 9, 10, 11, 12, 13, 14, 15, 16, 17, 18, 19, 20, 21, 22, 23, 24, 25, 26, 27]). Detailed information regarding the patients and procedures are listed in Table 2 (Ref. [5, 6, 9, 10, 11, 12, 13, 14, 15, 16, 17, 18, 19, 20, 21, 22, 23, 24, 25, 26, 27]).

**Table 1.** **General characteristics of the enrolled studies**.

Study	Country/Territory	Center	Data from	Study period	Follow-up period	Study type
Chen, 2019 [5]	6 countries	27 centers	DKCRUSH-V registry	Dec. 2011–Feb. 2016	1,2 and 3 years	RCT
Hildick-Smith, 2021 [6]	11 European countries	31 centers	EBC MAIN registry	Feb. 2016–Nov. 2019	1 year	RCT
Gao, 2015 [11]	China	1 center	Local database	Jan. 2004–Dec. 2010	4 years	Non-RCT
Kawamoto, 2018 [25]	Europe and Japanese	6 centers	FAILS 2 registry	Jul. 2006–Mar. 2015	1 year, 3 years	Non-RCT
Kim, 2010 [14]	Korea	12 centers	MAIN-COMPARE registry	May 2003–Jun. 2006	3 years	Non-RCT
Palmerini, 2008 [17]	Italy	19 centers	Local database, GISE-SICI registry	Jan. 2002–Dec. 2006	2 years	Non-RCT
Valgimigli, 2006 [27]	Netherlands	1 center	REAEARCH, T-SEARCH registry	Apr. 2002–Jun. 2004	587 days	Non-RCT
Zhang, 2015 [21]	China	-	Local database	May 2009–May 2013	1 year	Non-RCT
Sarma, 2021 [9]	Italy	1 center	Local database	Apr. 2013–Jul. 2018	2 years	Non-RCT
Lee, 2020 [13]	International	Multi-centers	IRIS-DES, IRIS-MAIN registry	May 2003–Jul. 2015	3.5 years	Non-RCT
Choi, 2020 [24]	Korea	21 centers	COBIS III registry	Jan. 2010–Dec. 2014	53 months	Non-RCT
Ferenc, 2018 [19]	Germany	-	BBK registry	Jan. 2004–Dec. 2014	3.1 years	Non-RCT
Cho, 2018 [18]	Korea	8+16 centers	KOMATE, COBIS II registry	Feb. 2002–Sep. 2013	25.9 months	Non-RCT
Kandzari, 2018 [22]	International	Multi-centers	EXCEL registry	-	3 years	Non-RCT
Rigatelli, 2022 [16]	Italy	1 center	Local database	Jan. 2008– May 2018	37.1 months	Non-RCT
Chen, 2012 [15]	China	1 center	Local database	Mar. 2004–Apr. 2007	5 years	Non-RCT
Kim, 2006 [12]	Korea	-	Local database	Mar. 2003–Nov. 2004	18 months	Non-RCT
Migliorini, 2017 [10]	Italy	1 center	Florence ULMD PCI registry	May 2008–Jul. 2015	1 years	Non-RCT
D’Ascenzo, 2016 [26]	Europe	9 centers	Local database	2002–2004	10 years	Non-RCT
Nasir, 2020 [23]	Pakistan	1 center	Local database	Jan. 2017 to Apr. 2018	6 months	Non-RCT
Alasmari, 2022 [20]	3 Gulf Countries	-	Gulf Left Main Registry	Jan. 2015 to Dec. 2019	20 months	Non-RCT

RCT, randomized control trial.

**Table 2.** **Baseline information of the enrolled patients and procedure**.

Study	Intervention	Sample size	Age, year	Male, %	DM, %	Hypertension, %	Dyslipidaemia, %
Chen, 2019 [5]	DK-Crush vs. PS	242/240	64/65	77.7/82.9	25.6/28.8	64.5/72.9	47.5/47.5
Hildick-Smith, 2021 [6]	PS vs. Culotte, DK-minicrush, T or TAP vs. PS	230/237	70.8/71.4	79/74	29/27	79/82	70/72
Gao, 2015 [11]	PS vs. DK-Crush, Classic crush, T, V, SKS vs. PS	661/372	60/60	81.1/81.7	22.7/26.3	55.4/56.7	49.5/49.5
Kawamoto, 2018 [25]	PS vs. Culotte, Crush, Mini-crush, T, V vs. PS	216/161	70.8/70.4	78.7/79.5	46.7/38.6	83.8/78.3	69.8/66.2
Kim, 2010 [14]	PS vs. Culotte, Crush, Kissing, T, V vs. PS	234/158	71.3/71.2	72.6/76.6	36.5/29.1	54.7/56.1	35.8/35.9
Palmerini, 2008 [17]	PS vs. Culotte, Crush, T, V vs. PS	456/317	72/70	73.6/77.2	33.0/24.3	-	63.4/68.3
Valgimigli, 2006 [27]	-	48/46	64/63	67/60	25/28	58/69	61/70
Zhang, 2015 [21]	PS vs. Culotte, Mini-crush, T, V vs. PS	50/38	56.8/62.1	68.0/73.7	14.0/15.8	64.0/78.9	20.0/28.9
Sarma, 2021 [9]	PS vs. T, TAP, DK-Crush, culotte, crush, mini crush vs. PS	56/11	57.77/60.90	71/81	48/81	57/72	-/18
Lee, 2020 [13]	-	440/562	64.4/64.4	77.3/77.9	39.1/35.2	63.6/64.2	14.5/9.4
Choi, 2020 [24]	PS vs. Culotte, Crush, Kissing, T, V, TAP, Kissing vs. PS	682/253	65.0/66.8	76.8/73.9	38.4/37.2	61.4/54.5	41.1/32.0
Ferenc, 2018 [19]	PS vs. Culotte, TAP vs. PS	477/390	70.6/70.2	74.8/74.6	29.4/28.5	84.7/83.6	-
Cho, 2018 [18]	PS vs. Culotte, Crush, Kissing, T, V, Kissing vs. PS	951/381	-	74.6/72.6	34.1/30.7	60.1/59.9	46.3/37.7
Kandzari, 2018 [22]	PS vs. T, modified T, TAP, Culotte, Crush, mini-crush, V, Kissing	344/185	66.2/66.8	79.9/76.2	28.8/34.6	73.8/76.2	73.0/70.1
Rigatelli, 2022 [16]	PS vs. Culotte, TAP, Nano-inverted-T vs. PS	171/396	-	53.2/56.8	28.1/20.5	55.6/44.2	40.9/33.8
Chen, 2012 [15]	PS vs. DK-Crush, culotte, T, Kissing, Crush vs. PS	232/401	67.7/66.7	79.3/79.6	29.7/27.4	76.7/70.1	51.3/53.9
Kim, 2006 [12]	PS vs. Kissing, Crush vs. PS	69/49	59.6/60.6	71.6/77.6	35.8/22.4	50.7/34.7	25.4/16.3
Migliorini, 2017 [10]	PS vs. Crush vs. PS	278/127	72/70	79/82	21/35	66/69	54/59
D’Ascenzo, 2016 [26]	PS vs. T, Crush vs. PS	174/85	66/65	79/79	43/36	73/71	72/77
Nasir, 2020 [23]	PS vs. DK-Crush, mini-crush, culotte and T	73/30	64.0/61.5	72.6/93.3	43.8/50	43.8/26.7	-
Alasmari, 2022 [20]	PS vs. Culotte, DK-Crush	173/1049	62.30/65.85	78.0/72.4	59.0/66.9	68.6/71.6	64.5/68.7

DM, diabetes mellitus; PS, provisional stenting; TAP, T stenting and small protrusion technique; SKS, simultaneous kissing stents technique; DK, double kissing technique; T, T stenting technique; V, V stenting technique; MI, myocardial infarction; PCI, percutaneous coronary intervention; IVUS, intravascular ultrasound; ACS, acute coronary syndromes.

**Table 2.** **Continued**.

Renal impairment, %	Prior MI, %	Current smoker, %	Prior PCI, %	Prior stroke, %	Peripheral vascular disease, %	IVUS	Stent type, %
-	21.1/21.7	-	-	-	-	-	2nd generation 100/100
5/4	26/28	16/13	41/43	7/7	14/16	36/31	zotarolimus 100/100
-	24.2/25.8	28.0/27.7	20.9/28.2	6.5/6.5	4.8/6.2	32.2/53.8	sirolimus 65.0/64.9
							paclitaxel 13.9/23.8
							2nd generation 21.1/11.4
48.7/47.4	37.4/28.1	12.3/18.2	53.0/51.0	6.3/8.3	-	22.2/27.3	biolimus 11.6/18.6
							everolimus 79.2/67.1
							zotarlolimus 7.9/11.2
							others 1.4/2.5
3.0/4.5	10.8/10.8	23.9/18.4	-	-	2.2/2.5	-	sirolimus and paclitaxel 100/100
11.4/10.8	-	38.5/34.7	-	-	25.6/18.9	-	-
-	40/39	17/22	37/24	-	-	-	sirolimus and paclitaxel 100/100
-	10.0/11.1	26.0/26.3	-	-	-	6.0/5.2	-
-	37/28	14/9	-	-	-	14/4	xience 80/90
							vascular concepts 16/9
4.5/4.3	6.6/8.9	28.0/24.0	17.5/21.5	8.0/7.5	2.5/3.6	-	1st generation 22.5/27.8
							2nd generation 72.5/72.2
5.6/3.6	5.1/5.1	25.2/21.3	16.7/17.8	-	-	62.6/68.0	everolimus 53.8/51.8
							zotarolimus 24.0/27.3
							biolimus 19.4/15.8
							mixed or other 2.8/5.1
-	26.0/23.6	11.7/12.3	32.5/28.5	-	-	-	sirolimus 15.7/23.3
							paclitaxel 13.2/12.3
							zotarolimus 28.5/26.9
							everolimus 38.8/33.8
4.2/4.1	-	34.6/26.9	18.8/25.3	-	-	54.6/62.8	1st generation 52.3/74.4
							2nd generation 47.7/25.6
-	19.2/20.8	64.8/64.1	20.1/22.8	4.7/8.1	-	-	everolimus 100/100
15.8/13.1	-	31.6/24.2	-	26.9/22.0	-	-	2nd generation 100/100
-	17.7/15.0	30.6/29.9	34.0/29.2	6.9/7.7	-	15.1/20.4	sirolimus or paclitaxel 100/100
-	-	19.4/30.6	11.9/18.4	-	-	89.6/87.8	-
-	22/23	-	-	-	-	64/82	xience 100/100
-	-	30/21	35/21	-	-	-	-
-	-	11/23.3	-	-	-	11.0/23.3	-
15.0/27.6	25.4/35.7	36.4/39.7	-	-	6.4/16.5	52/28.4	everolimus 83.2/88.5
							zotarolimus 25.4/26.3
							sirolimus 10.4/5.7
							biolimus 3.5/4.3
							others 1.3/1.0

**Table 2.** **Continued**.

SYNTAX score, %	Medina classification, %	Double stenting type, %	Duration of dual antiplatelet therapy
-	-	DK-Crush 100	100 mg/day aspirin and clopi-dogrel, 75 mg/day for at least 12 months.
0–22 30/26	1,1,1 90/89	culotte 53	Aspirin 75 mg daily was continued long term. Clopidogrel 75 mg daily was given for a minimum of 6 months.
22–32 56/57	0,1,1 10/11	DK-Crush 5	
missing 15/17		T or TAP 32	
		unstated 4	
		missing data 3	
-	-	crush 69.1	300 mg daily for 3 months and followed by 100 mg daily in definitely.
		T 14.0	
		V or SKS 12.1	
		culotte 4.8	
low score 26.8/23.5	0,1,1 10.6/14.9	crush 7.5	-
intermediate score 35.4/37.3	1,0,1 15.7/12.4	colotte 32.9	
high score 37.9/39.2	1,1,1 73.6/72.7	mini-crush 39.8	
		T 14.3	
		V 5.6	
mean score 23.5/27.0	-	crush 45.6	After the procedure, aspirin was continued indefinitely and clopidogrel was continued for at least 6 months.
		kissing 34.8	
		T 15.8	
		V 2.5	
		culotte 1.3	
-	-	T 40.7	-
		V 19.1	
		culotte 1.6	
		crush 38.6	
-	-	-	all patients were maintain aspirin lifelong, clopidogrel was prescribed for 6 months in both groups.
-	1,1,1 4/55.3	mini-crush 50.0	all patients received 300 mg/day aspirin for one month. Thereafter, they received 100 mg/day indefinitely for life. Clopidogrel (75 mg/d) was continued for at least 12 months.
	1,0,1 2/2.6	culotte 36.8	
	0,1,1 2/18.4	T 7.9	
		V 5.3	
-	1,1,1 33/54	T 18	-
	1,1,0 35/9	TAP 9	
	1,0,1 0/9	DK-Crush 54	
	0,1,1 1/9	culotte 18	
	0,0,1 0/0	crush/mini crush 0	
	0,1,0 28/18		
	1,0,0 0/0		
-	1,1,1 93.6/93.4	-	After the procedure, aspirin was continued indefinitely and P2Y12 inhibitors were prescribed for at least 12 months.
	0,1,1 6.4/6.6		
-	1,1,1 13.6/50.6	crush 56.1	100 mg of aspirin was continued indefinitely, and the maintenance duration of clopidogrel (75 mg/day), prasugrel (10 mg/day), or ticagrelor (90 mg twice daily) were also at the operators’ discretion.
	1,0,1 2.8/7.5	T or TAP 23.7	
	0,1,1 4.3/17.4	culotte 6.3	
	1,0,0 11.3/2.8	kissing or V 10.3	
	1,1,0 24.0/5.9	others 3.6	
	0,1,0 40.3/3.6		
	0,0,1 3.7/12.3		
-	1,1,1 30.4/60.3	culotte 10.8	Post-PCI, we recommended lifelong aspirin (≥100 mg per day) and clopidogrel (≥75 mg per day) or prasugrel or ticagrelor for 6 or 12 months.
	1,1,0 33.8/7.9	TAP 88.2	
	1,0,1 9.9/12.8		
	1,0,0 14.3/2.8		
	0,1,1 2.1/10.3		
	0,1,0 8.2/2.3		
	0,0,1 1.5/3.6		
-	1,1,1 21.6/51.9	T 34.9	Aspirin was continued indefinitely, and clopidogrel duration was left to the operator’s discretion.
	1,0,1 5.1/7.8	Crush 42.4	
	0,1,1 2.3/13.4	kissing or V 3.4	
	1,0,0 13.5/2.1	culotte 7.1	
	1,1,0 31.2/9.8	others 2.3	
	0,1,0 24.1/6.5		
	0,0,1 2.3/8.5		
0–22 29.1/17.3	1,0,0 31.0/7.6	T, modified T or TAP 50.8	-
23–32 42.9/44.1	0,1,0 4.3/25	culotte 23.2	
≥33 27.9/38.5	1,1,0 30.0/11.0	crush or mini-crush 14.4	
	0,0,1 0/1.7	V 6.1	
	1,0,1 12.4/18.0	kissing 2.8	
	0,1,1 0.5/4.2	others 2.8	
	1,1,1 21.4/54.2		
-	1,1,1 43.3/34.8	-	Twelve-month Ticagrelor or Prasugrel treatment in case of ACS patients or 12-month Clopidogrel 75 mg in the other cases and life-long aspirin were recommended to all patients according to our regional guidelines.
	0,1,1 29.8/24.5		
mean score 39.2/34.5	0,1,1 24.7/27.4	DK-Crush 38.9	300 mg daily for 3 months and followed by 100 mg daily in definitely.
	1,1,1 56.5/63.6	others 61.3	
	1,0,1 4.7/4.8		
-	-	-	All patients received aspirin (200 mg/day) indefinitely and a loading dose of 300 mg of clopidogrel followed by a single 75 mg/day dose for 6 months. In addition, 200 mg of cilostazol was administered as a loading dose, followed by 100 mg 2 times daily for 1 month.
≥33 47/50	1,1,1 4/100	-	Chronic antithrombotic treatment included aspirin (300 mg/day indefinitely) and clopidogrel 75 mg daily or prasugrel 10 mg daily for at least 1 year.
	1,0,0 4/0		
	1,1,0 69/0		
	1,0,1 23/0		
median values:22 ± 8/27 ± 9	-	-	All patients were prescribed lifelong aspirin 75 mg once daily for life and clopidogrel 75 mg for 6–12 months or longer.
first tertile 54/43			
second tertile 34/34			
third tertile 12/27			
<22 21.9/0.0	1,1,1 45.2/100	DK-Crush 0	Post-PCI, 300 mg/day of aspirin was prescribed to all patients for one month, which was reduced to 75 mg/day to be continued indefinitely thereafter. In addition, they received clopidogrel 300 mg in divided doses for the first month, later reduced to 75 mg/day for at least one year after the PCI.
22–33 76.7/70.0	1,1,0 47.9/0	Mini crush 53.3	
>33 1.4/30.0	0,1,1 0/0	SKS 13.3	
	1,0,1 6.8/0	Culotte 0	
		T stenting 16.7	
		Other two-stent 3.3	
		modified techniques 13.3	
low (≤22) 41.0/22.2	1,1,1 13/16.9	Double kissing crush/standard crush 76.0	-
intermediate (23–32) 42.2/58.0	1,1,0 39.13/16.74	Mini-crush 1.4	
high (≥33) 16.8/19.8	1,0,1 6.1/4.6	Culotte 14.0	
mean score:7.70/7.76	0,1,1 23.5/23.4	T-stenting 1.1	
	1,0,0 4.3/1.3	V-stenting 1.1	
	0,1,0 13/4.1	T and small protrusion 6.3	
	0,0,1 0.9/1.12		

### 3.2 Quality Assessment of the Studies

**Fig. 2.**
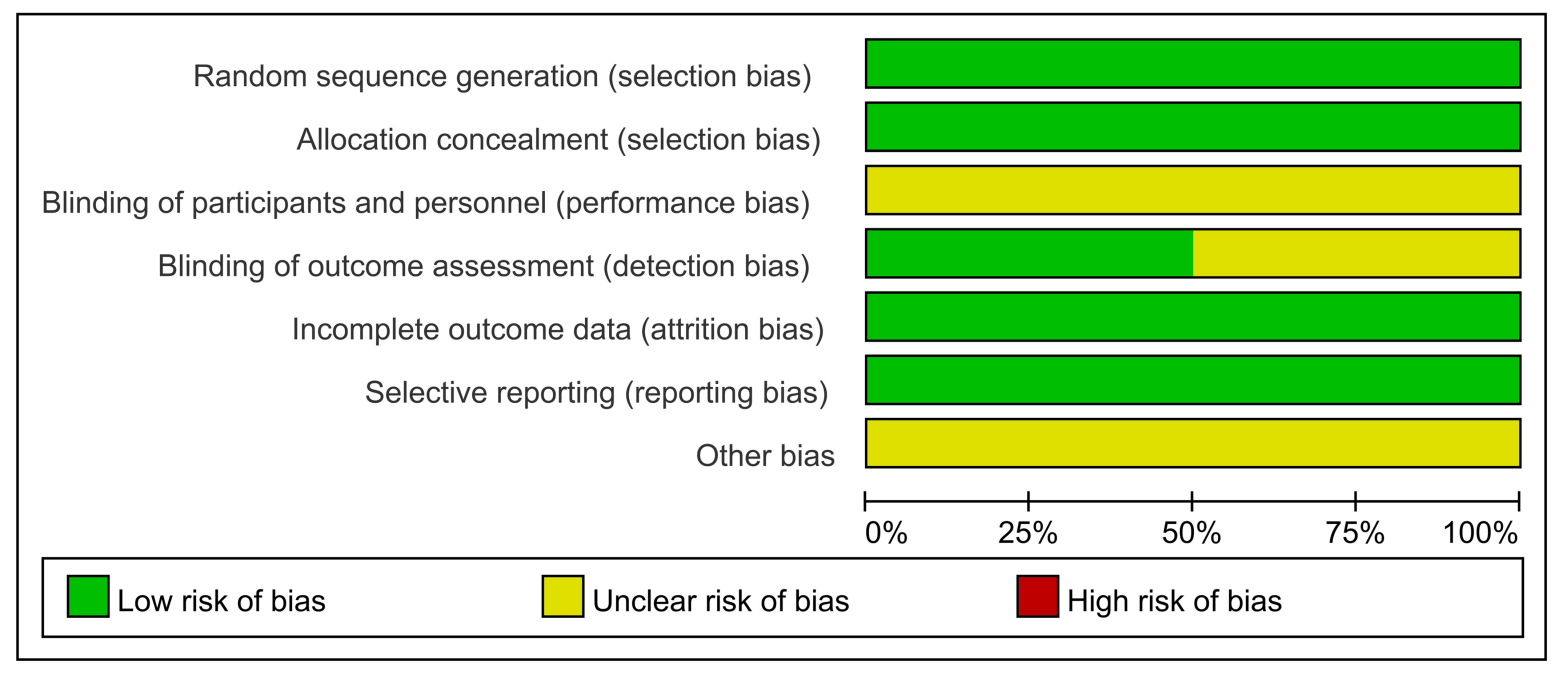
**Quality assessment of the RCTs with Cochrane Collaboration’s tool**. RCTs, randomized controlled trails.

The quality of the RCTs was evaluated using the Cochrane Collaboration tool. The seven domains of the two RCTs are all displayed in Fig. 2. The quality of observational studies was assessed using NOS. All 19 studies were considered to have a low risk of bias (Table 3, Ref. [9, 10, 11, 12, 13, 14, 15, 16, 17, 18, 19, 20, 21, 22, 23, 24, 25, 26, 27]).

**Table 3.** **Quality assessment of the cohort studies by NOS**.

Study	Selection	Comparability	Outcome	Total score
Gao, 2015 [11]	☆☆☆☆	☆☆	☆☆	8
Kawamoto, 2018 [25]	☆☆☆	☆☆	☆☆	7
Kim, 2010 [14]	☆☆☆	☆☆	☆☆	7
Palmerini, 2008 [17]	☆☆☆	☆☆	☆☆	7
Valgimigli, 2006 [27]	☆☆☆	☆☆	☆☆	7
Zhang, 2015 [21]	☆☆☆	☆☆	☆☆☆	8
Sarma, 2021 [9]	☆☆☆	☆☆	☆☆	7
Lee, 2020 [13]	☆☆☆	☆☆	☆☆	7
Choi, 2020 [24]	☆☆☆	☆☆	☆☆	7
Ferenc, 2018 [19]	☆☆☆	☆☆	☆☆	7
Cho, 2018 [18]	☆☆☆	☆☆	☆☆	7
Kandzari, 2018 [22]	☆☆☆	☆☆	☆☆	7
Rigatelli, 2022 [16]	☆☆☆	☆☆	☆☆	7
Chen, 2012 [15]	☆☆☆☆	☆☆	☆☆	7
Kim, 2006 [12]	☆☆☆	☆☆	☆☆	7
Alasmari, 2022 [20]	☆☆☆	☆☆	☆☆	7
D’Ascenzo, 2016 [26]	☆☆☆	☆☆	☆☆	7
Nasir, 2020 [23]	☆☆☆	☆☆	☆☆	7
Migliorini, 2017 [10]	☆☆☆☆	☆☆	☆☆	7

NOS, Newcastle-Ottawa Quality Assessment Scale.

### 3.3 Primary Endpoint

#### Major Adverse Cardiac Events

**Fig. 3.**
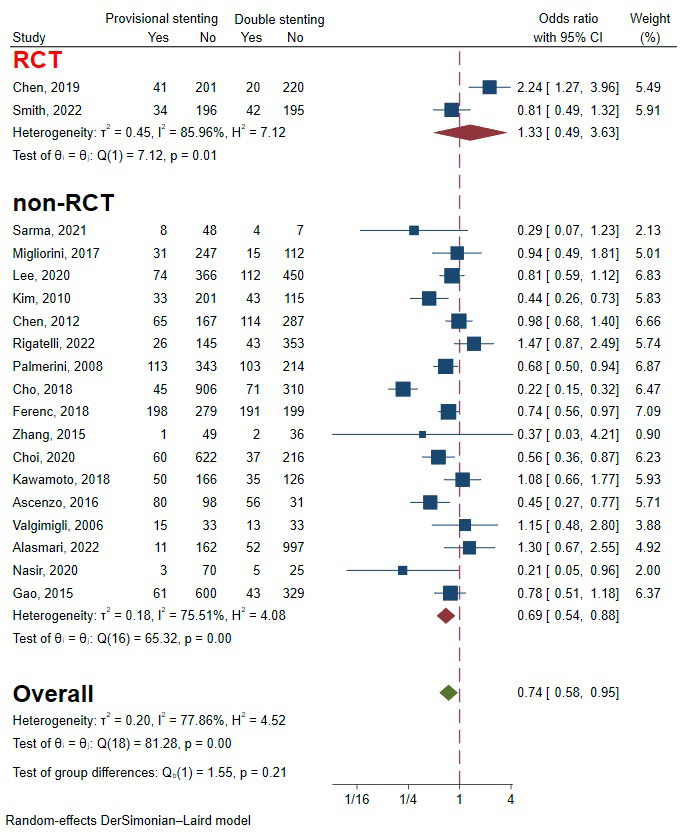
**Forest plot of comparisons of major adverse cardiac events between provisional stenting and double stenting**. RCT, randomized controlled trail.

**Fig. 4.**
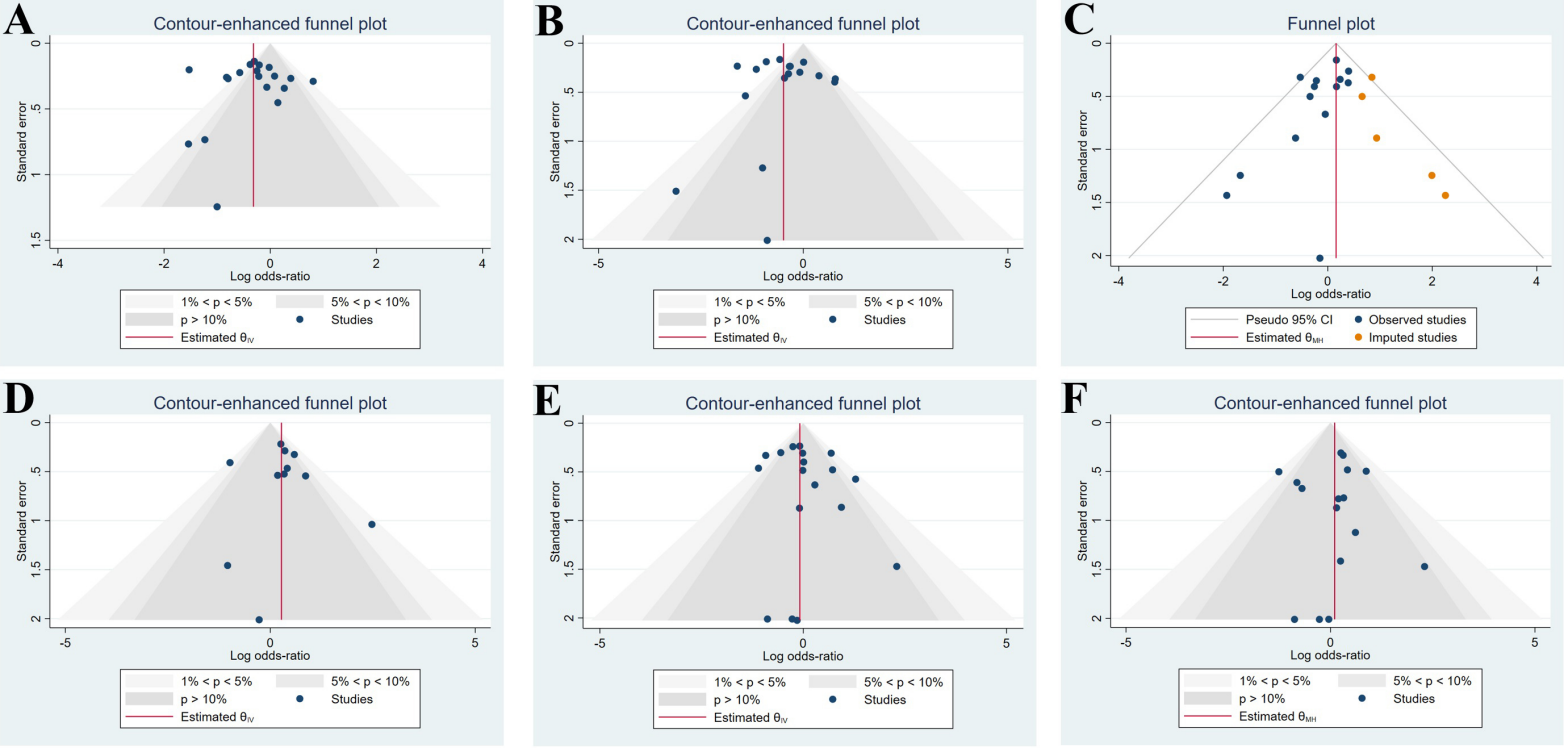
**Contour-enhanced funnel plot for publication bias evaluation of studies concerning major adverse cardiac events (A), target lesion revascularization (B), all-cause death (C), cardiac death (D), myocardial infarction (E), stent thrombosis (F)**.

**Fig. 5.**
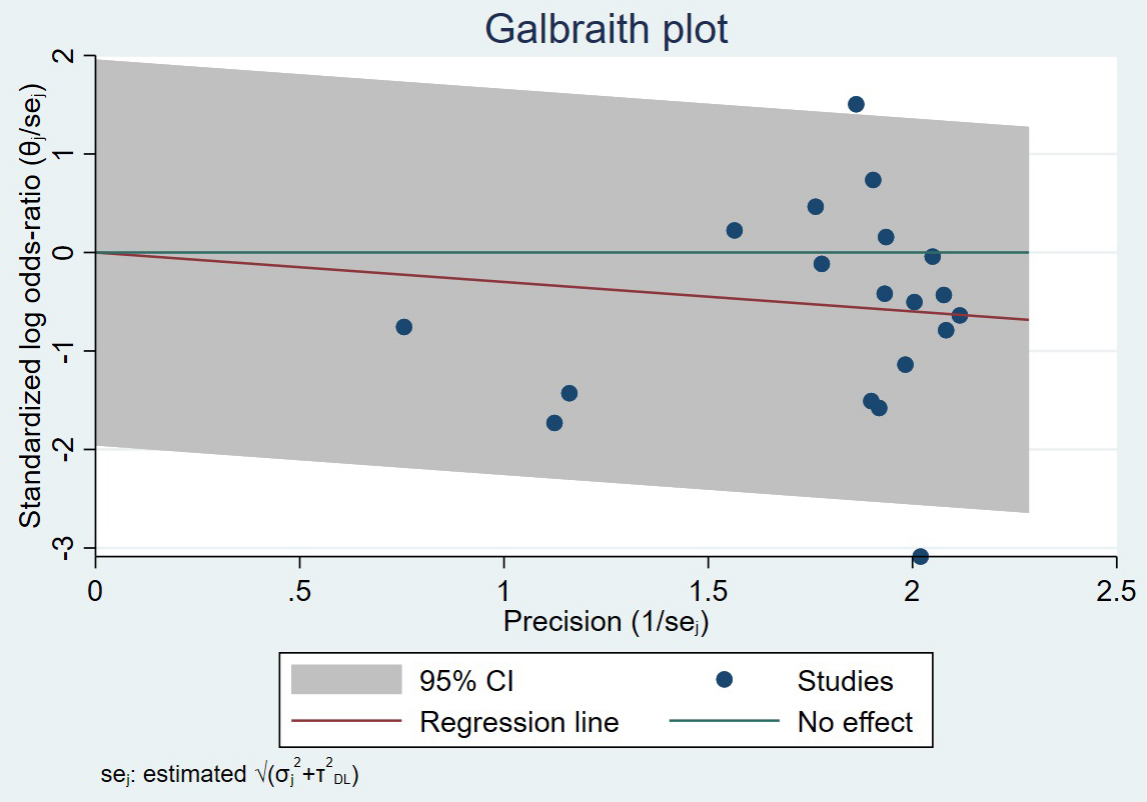
**Galbraith plot for Heterogeneity test of studies concerning major adverse cardiac events**.

**Fig. 6.**
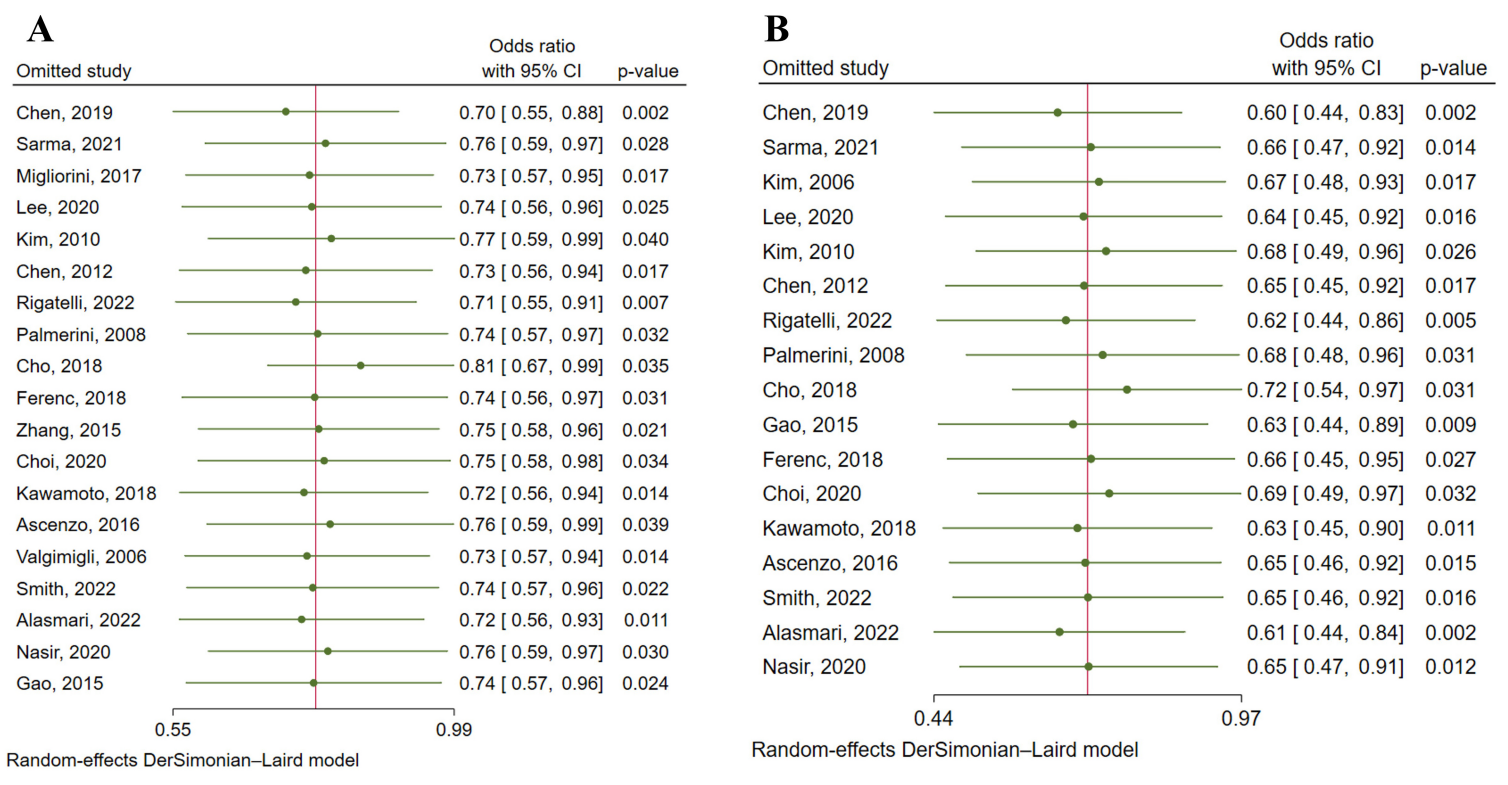
**Sensitivity analysis of the heterogeneity of studies concerning major adverse cardiac events (A) and target lesion revascularization (B)**.

Testing for the overall effect of the two RCTs and 17 observational studies [5, 6, 9, 10, 11, 13, 14, 15, 16, 17, 18, 19, 20, 21, 23, 24, 25, 26, 27], including 10,805 patients, revealed that the provisional stenting strategy was significantly superior to double stenting. The heterogeneity was relatively large ($I^{2}$ = 77.89%, *p* = 0.00), so a random-effects model was used (Fig. 3). Funnel plots and regression-based Egger test showed no evident publication bias (*p* = 0.39) (Fig. 4A). Heterogeneity test and sensitivity analysis pointed out that heterogeneity mainly came from two studies [5, 24] (Fig. 5 and Fig. 6A). After eliminating these two studies, the heterogeneity was significantly reduced, and the subsequent result was consistent with the primary one (**Supplementary Fig. 1**). Subgroup analysis of study types drew opposite conclusions, but the difference wasn’t statistically significant (Fig. 3) (*p* = 0.21).

### 3.4 Secondary Endpoints

#### 3.4.1 Target Lesion Revascularization

**Fig. 7.**
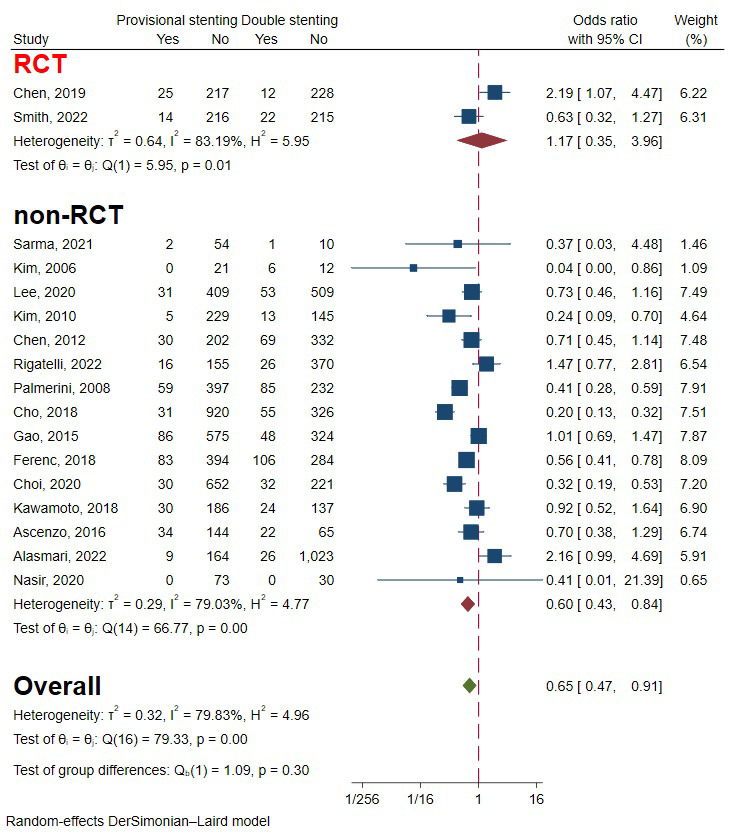
**Forest plot of comparisons of target lesion revascularization between provisional stenting and double stenting**. RCT, randomized controlled trail.

The results of TLR were similar to those of MACE. A total of two RCTs and 15 observational studies involving 10,556 patients were analysed [5, 6, 9, 11, 12, 13, 14, 15, 16, 17, 18, 19, 20, 23, 24, 25, 26]. The overall effect favoured provisional stenting for significantly lower TLR. The heterogeneity was relatively large ($I^{2}$ = 79.83%, *p* < 0.001), so a random-effects model was used (Fig. 7). Funnel plots and regression-based Egger test showed no evident publication bias (*p* = 0.35) (Fig. 4B). Sensitivity analysis pointed out that heterogeneity mainly came from studies of Chen [5], Cho [18] and Alasmari [20] (Fig. 6B). After eliminating these studies, the heterogeneity was reduced and the result was consistent with the primary result (**Supplementary Fig. 2**). Subgroup analysis of study type drew opposite conclusions, but the difference wasn’t statistically significant (Fig. 7) (*p* = 0.30).

#### 3.4.2 Target Vessel Revascularization

**Fig. 8.**
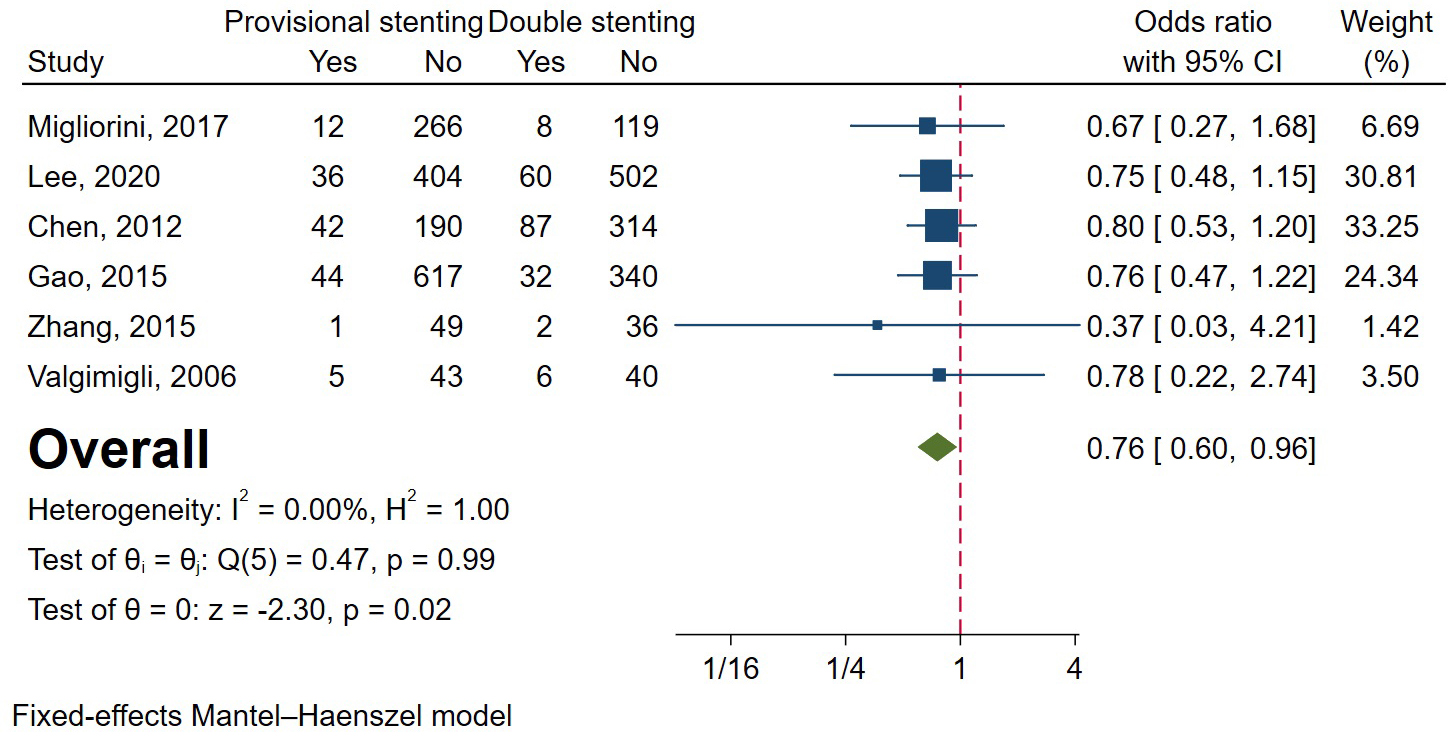
**Forest plot of comparisons of target vessel revascularization between provisional stenting and double stenting**.

Six observational studies involving 3255 enrolled patients were analysed for occurrence of TVR [10, 11, 13, 15, 21, 27]. The heterogeneity was pretty small ($I^{2}$ = 0%, *p* = 0.99), so a fixed-effects model was used. The overall effect revealed that provisional stenting had a significantly lower TVR than double stenting (Fig. 8).

### 3.5 All-Cause Death

**Fig. 9.**
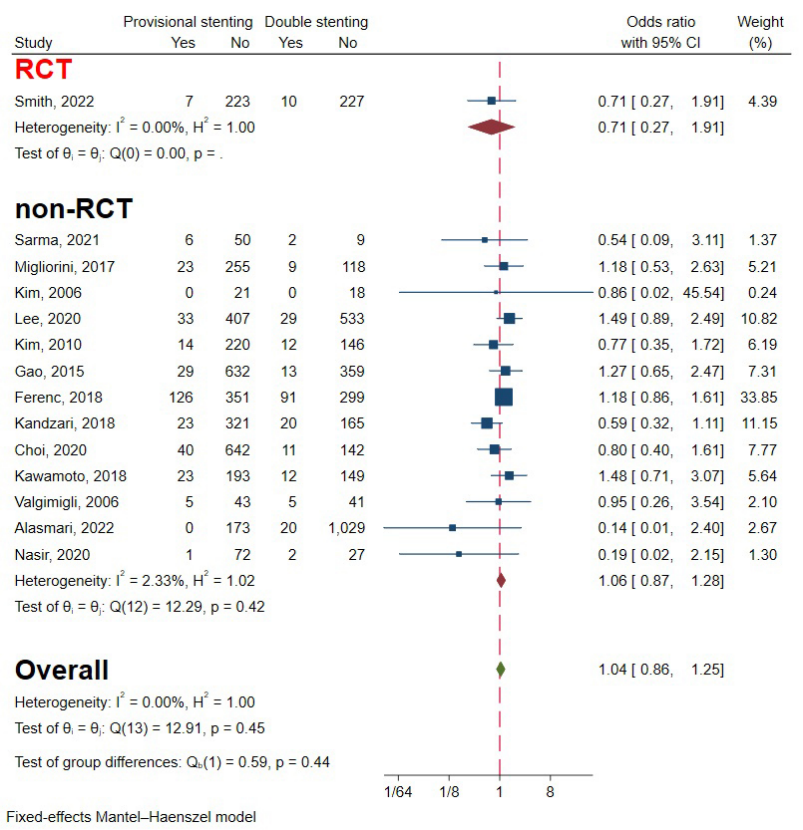
**Forest plot of comparisons of all-cause death between provisional stenting and double stenting**. RCT, randomized controlled trail.

One RCT and 13 observational studies involving 7532 patients were included to evaluate the occurrence of all-cause death [6, 9, 10, 11, 12, 13, 14, 19, 20, 22, 23, 24, 25, 27]. Analysis was favourable for double stenting for lower all-cause death incidence, but the difference wasn’t significant. The heterogeneity was reasonably small, so a fixed-effects model was used ($I^{2}$ = 0%, *p* = 0.45) (Fig. 9). Funnel plots and regression-based Egger test showed evident publication bias (*p* = 0.04). A non-parametric trim-and-fill analysis of publication bias was performed, and the results demonstrated that five studies should be imputed to the right side (Fig. 4C). After imputation, the aggregated OR value was enlarged from 1.052 [0.872, 1.270] to 1.173 [0.984, 1.398], but there was still no significant difference. Subgroup analysis of study type drew opposite conclusions, but the difference wasn’t statically significant (Fig. 9) (*p* = 0.44).

### 3.6 Cardiac Death

**Fig. 10.**
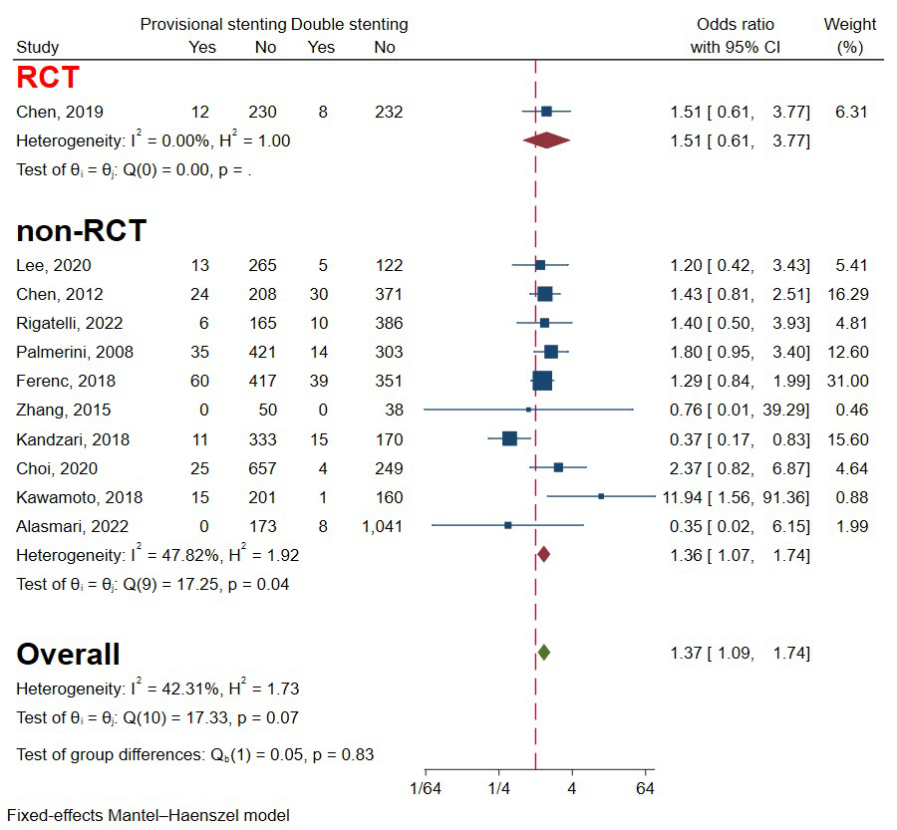
**Forest plot of comparisons of cardiac death between provisional stenting and double stenting**. RCT, randomized controlled trail.

One RCT and 10 observational studies involving 6878 patients were included to evaluate the occurrence of cardiac death [5, 13, 15, 16, 17, 19, 20, 21, 22, 24, 25]. The analysis was favourable for double stenting for significantly lower cardiac death. The heterogeneity was acceptable, so a fixed-effects model was used ($I^{2}$ = 42.31%, *p* = 0.07) (Fig. 10). Funnel plots and a regression-based Egger test showed no evident publication bias (*p* = 0.80) (Fig. 4D). Subgroup analysis drew consistent conclusions between RCTs and non-RCTs (*p* = 0.83) (Fig. 10).

### 3.7 Myocardial Infarction

**Fig. 11.**
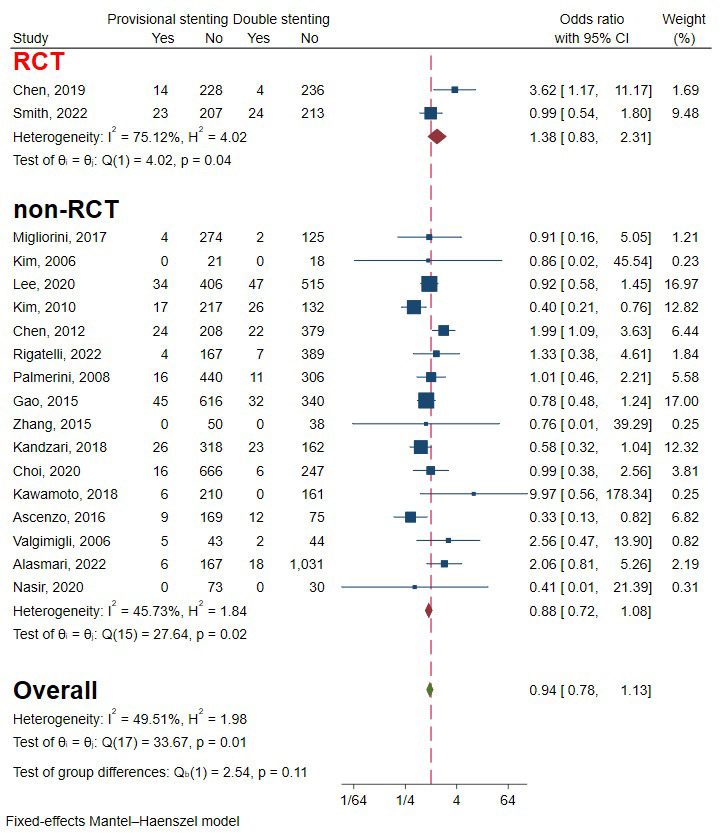
**Forest plot of comparisons of myocardial infarction between provisional stenting and double stenting**. RCT, randomized controlled trail.

Two RCTs and 16 observational studies involving 9406 patients were included to evaluate the occurrence of MI [5, 6, 10, 11, 12, 13, 14, 15, 16, 17, 20, 21, 22, 23, 24, 25, 26, 27]. The overall effect showed there was no significant difference between provisional stenting and double stenting. The heterogeneity mainly came from subgroups of RCTs. The overall heterogeneity was acceptable, so a fixed-effect model was used ($I^{2}$ = 49.51%, *p* = 0.01) (Fig. 11). Funnel plots and regression-based Egger test showed no evident publication bias (*p* = 0.30) (Fig. 4E). Subgroup analysis of study type drawn opposite conclusions, but the difference wasn’t statically significant (Fig. 11) (*p* = 0.11).

### 3.8 Stent Thrombosis

**Fig. 12.**
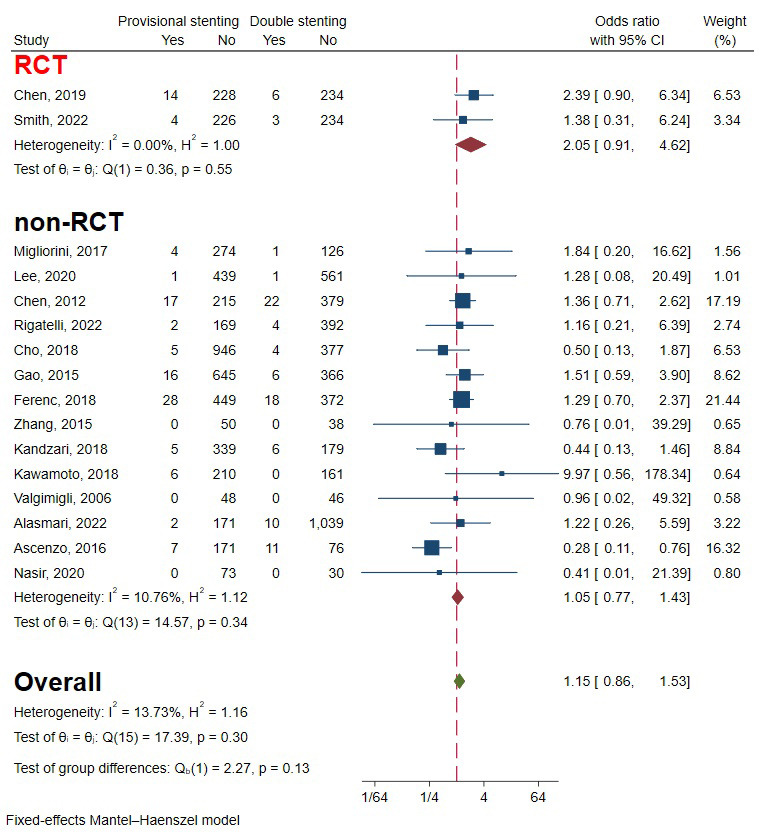
**Forest plot of comparisons of stent thrombosis between provisional stenting and double stenting**. RCT, randomized controlled trail.

Two RCTs and 14 observational studies involving 9466 patients were included to evaluate the occurrence of ST [5, 6, 10, 11, 13, 15, 16, 18, 19, 20, 21, 22, 23, 25, 26, 27]. The overall effect showed there was no significant difference between provisional stenting and double stenting. The heterogeneity was relatively small, so a fixed-effect model was used ($I^{2}$ = 13.73%, *p* = 0.30) (Fig. 12). Funnel plots and a regression-based Egger test showed no evident publication bias (*p* = 0.87) (Fig. 4F). Subgroup analysis drew consistent conclusion of favoring double stenting between RCTs and non-RCTs (*p* = 0.13) (Fig. 12).

## 4. Discussion

A total of two RCTs and 19 observational studies were included in this study [5, 6, 9, 10, 11, 12, 13, 14, 15, 16, 17, 18, 19, 20, 21, 22, 23, 24, 25, 26, 27]. For the endpoints of MACE and TLR, the heterogeneity was relatively large, and it mainly came from the RCT subgroup. We only identified two RCTs, but they drew conflicting conclusions concerning MACE, TLR and MI, although the difference did not reach statistical significance. We believe that the heterogeneity of the two RCTs may be due to the different techniques of double stenting. In the study from Chen [5], only DK-Crush was performed for double stenting, while in the study from Hildick-Smith [6], a composition of Culotte, DK-minicrush, T or T stenting and small protrusion technique (TAP) was performed. This reminded us that DK-Crush was likely better than provisional stenting, while provisional stenting was better than other double stenting.

Subgroup analysis of RCT and non-RCT revealed that the two aggregated OR were opposing in MACE, TLR, all-cause death and MI occurrences, and consistent in cardiac death and ST occurrences. Though RCTs have a higher level of evidence than in observational studies, their small size became the greatest limitation for this review.

We identified publication bias only when analysing all-cause death occurrence. We performed a non-parametric trim-and-fill analysis for the publication bias. After virtually imputing five studies, the funnel plot became symmetric, and the bias was reduced. The adjusted OR value was enlarged from 1.052 [0.872, 1.270] to 1.173 [0.984, 1.398]. However, the results still favoured the double stenting strategy.

The aggregated OR values of all endpoints are displayed in Table 4. Our analysis revealed that provisional stenting had a significantly lower incidence of MACE, mainly driven by TLR and TVR and double stenting had a significantly lower incidence of cardiac death. Additionally, provisional stenting tended to have a lower occurrence of MI, while double stenting tended to have a lower occurrence of all-cause death and ST. From these results, it was hard for us to conclude which performed better. Considering the importance of survival, double stenting might be more recommended.

**Table 4.** **Summarize of the aggregated OR values of all endpoints**.

		Aggregated OR (RCT)	Aggregated OR (non-RCT)	Aggregated OR (Overall)
Primary endpoint				
	MACE	1.33	0.69*	0.74*
Secondary endpoints				
	TLR	1.17	0.60*	0.65*
	TVR	-	0.76*	0.76*
	All-cause death	0.71	1.06	1.04
	Cardiac death	1.51	1.36*	1.37*
	MI	1.38	0.88	0.94
	ST	2.05	1.05	1.15

*, *p* < 0.05 (Provisional stenting vs. Double stenting). MACE, major adverse cardiac events; TLR, target lesion revascularization; TVR, target vessel revascularization; MI, myocardial infarction; ST, stent thrombosis; OR, odds ratio; RCT, randomized controlled trail.

The latest systematic review and meta-analysis comparing the two strategies for LM was published by Abdelfattah *et al*. [28], in which 12 studies of 7105 patients were included. In that review, only the 2nd generation of DES was considered. However, in our pre-analysis we found that DES type didn’t affect the OR value. So as to enlarge the sample size, we enrolled both the 1st and 2nd DES, and the sample size was nearly doubled. A recent large sample-sized study conducted by Alasmari in 2022 [20] was added in our review. The differences in outcomes between the two meta-analyses mainly lie in the occurrences of cardiac death and MI.

Vescovo *et al*. [29] published a network meta-analysis comparing different double stenting techniques and provisional stenting. Network meta-analysis was recommended to select a specific technique. However, detailed subdivisions reduced the sample size. As provisional stenting and double stenting were considered as two different strategies, rather than two different techniques, there was still a necessity to conduct this systematic review and meta-analysis to clarify which performed better. It could help operators make the optimal strategy when dealing with LM bifurcation lesions.

## 5. Limitations

The limitations of this study mainly lie in the definitions of endpoints that varied across studies, the double stenting techniques that varied across studies, the performance of IVUS, POT, and double balloon kissing (DBK) that varied across studies, and the long span of 2002 to 2019. At last, this review was not registered and a protocol was not prepared.

## 6. Conclusions

The provisional stenting strategy was associated with a significantly lower occurrence of MACE, mainly driven by TLR and TVR, but a higher occurrence of cardiac death. Further investigations are needed, especially those involving RCTs, to confirm which strategy performs better.

## Abbreviations

UPLMB, Unprotected left main distal bifurcation; LM, left main; RCTs, randomized controlled trials; TLF, target lesion revascularization failure ; ST, stent thrombosis ; MACE, major adverse cardiac events; DES, drug-eluting stent; MI, myocardial infarction; TVR, target vessel revascularization; TLR, target lesion revascularization; IVUS, intravascular ultrasound; POT, proximal optimal technique; PTCA, percutaneous transluminal coronary angioplasty; CABG, coronary artery bypass surgery ; ARC, Academic Research Consortium; NOS, Newcastle-Ottawa Quality Assessment Scale; OR, Odds ratio; CI, confidence interval.

## Supplementary Material

Supplementary material associated with this article can be found, in the online version, at https://doi.org/10.31083/j.rcm2408216.
